# Supplementary material for: From sequence to enzyme mechanism using multi-label machine learning
Source: BMC Bioinformatics. 2014 May 19;15:150. doi: 10.1186/1471-2105-15-150 (PMC4229970; doi:10.1186/1471-2105-15-150)
Supplement: Additional file 2 — Java code of ml2db. Additional file ml2db_code.tar.gz contains the Java source code to run the multi-label machine learning experiments and save the results to database. The code’s Javadoc is included. [file 1471-2105-15-150-S2.zip › additional file 2/ml2db/utils/doc/uk/ac/standrews/utils/main/database/package-use.html]

Uses of Package uk.ac.standrews.utils.main.database


JavaScript is disabled on your browser.


- Overview
- Package
- Class
- Use
- Tree
- Deprecated
- Index
- Help

- Prev
- Next

- Frames
- No Frames

- All Classes

# Uses of Package uk.ac.standrews.utils.main.database

- Packages that use uk.ac.standrews.utils.main.database

  | Package | Description |
  | --- | --- |
  |  |  |
  | --- | --- |
  | uk.ac.standrews.utils.main.database |  |
  | uk.ac.standrews.utils.main.setutils |  |
  | uk.ac.standrews.utils.test.database |  |
- Classes in uk.ac.standrews.utils.main.database used by uk.ac.standrews.utils.main.database

  Class and Description

  DbConn

  A DbConnectionProperties object contains the properties needed to connect to
  a database through JDBC.

  DbConnectionSingleton

  A singleton database connection

  DbCreator

  Class

  DbManaged

  Managed is a class which has a `DbManager` (an object that coordinates
  its database activities)

  DbManager

  A database manager provides object to create a database, write and read from
  it.

  DbManagerSingleton

  Class

  DbReader

  Class to read from a database.

  DbWriter

  Provides methods to write to a database.

  Manager

  A manager manages: for example, for a database object, the `DbManager`
  manages a `DbCreator` to create the tables, `DbReader` to read
  data from and `DbWriter` to write data to the database.

  Table

  An SQL database table definition (table name + columns)

  TableColumn

  A column object contains all the information to create, fill and delete a
  database table column

  TableCreator

  Class to create a table object in the database.

  TableManager

  Class to create/read/write to a database table.

  TableReader

  Class

  TableRow

  Contains the data for a generic row (a list of [table column, value] entries)
  to be written to a database table.

  TableWriter

  A TableWriter object contains all the information to create, fill and delete
  a database table.
- Classes in uk.ac.standrews.utils.main.database used by uk.ac.standrews.utils.main.setutils

  Class and Description

  DbManager

  A database manager provides object to create a database, write and read from
  it.
- Classes in uk.ac.standrews.utils.main.database used by uk.ac.standrews.utils.test.database

  Class and Description

  DbConn

  A DbConnectionProperties object contains the properties needed to connect to
  a database through JDBC.

  DbCreator

  Class

  DbManager

  A database manager provides object to create a database, write and read from
  it.

  DbReader

  Class to read from a database.

  DbWriter

  Provides methods to write to a database.

  Table

  An SQL database table definition (table name + columns)

  TableCreator

  Class to create a table object in the database.

  TableReader

  Class

  TableRow

  Contains the data for a generic row (a list of [table column, value] entries)
  to be written to a database table.

  TableWriter

  A TableWriter object contains all the information to create, fill and delete
  a database table.

- Overview
- Package
- Class
- Use
- Tree
- Deprecated
- Index
- Help

- Prev
- Next

- Frames
- No Frames

- All Classes
